# Exposure to Workplace Bullying: The Role of Coping Strategies in Dealing with Work Stressors

**DOI:** 10.1155/2017/1019529

**Published:** 2017-11-15

**Authors:** Whitney Van den Brande, Elfi Baillien, Tinne Vander Elst, Hans De Witte, Anja Van den Broeck, Lode Godderis

**Affiliations:** ^1^Research Group Occupational & Organisational Psychology and Professional Learning, KU Leuven, Leuven, Belgium; ^2^Research Centre for Work and Organisation Studies, KU Leuven, Leuven, Belgium; ^3^Knowledge, Information and Research Centre, IDEWE (External Service for Prevention and Protection at Work), Leuven, Belgium; ^4^Optentia Research Focus Area, North-West University, Vanderbijlpark, South Africa; ^5^Environment and Health, KU Leuven, Leuven, Belgium

## Abstract

Studies investigating both work- and individual-related antecedents of workplace bullying are scarce. In reply, this study investigated the interaction between workload, job insecurity, role conflict, and role ambiguity (i.e., work-related antecedents), and problem- and emotion-focused coping strategies (i.e., individual-related antecedents) in association with exposure to workplace bullying. Problem-focused coping strategies were hypothesised to decrease (i.e., buffer) the associations between workload, job insecurity, role conflict, and role ambiguity and exposure to bullying, while emotion-focused coping strategies were hypothesised to increase (i.e., amplify) these associations. Results for a heterogeneous sample (*N* = 3,105) did not provide evidence for problem-focused coping strategies as moderators. As expected, some emotion-focused coping strategies amplified the associations between work-related antecedents and bullying: employees using “focus on and venting of emotions” or “behavioural disengagement” in dealing with job insecurity, role conflict, or role ambiguity were more likely to be exposed to bullying. Similarly, “seeking social support for emotional reasons” and “mental disengagement” amplified the associations of role ambiguity and the associations of both role conflict and role ambiguity, respectively. To prevent bullying, organisations may train employees in tempering emotion-focused coping strategies, especially when experiencing job insecurity, role conflict, or role ambiguity.

## 1. Introduction

Workplace bullying is defined as the perceived situation in which an employee is systematically and repeatedly the* target *of work-related and/or personal negative acts at work [1]. Bullying has become an issue in many organisations. Prevalence rates range from 3% up to 15% in Europe [2], such that between 3% and 4% of European employees experience bullying behaviours weekly (i.e., serious bullying), while 9% to 15% experience bullying behaviours monthly (i.e., occasional bullying) [3]. As being exposed to workplace bullying is associated with health impairment—such as burnout [4], symptoms of posttraumatic stress disorder [5], and depression [6]—studies have investigated antecedents that may prevent bullying [2, 7].

To date, these studies have mainly focused on work-related antecedents that trigger exposure to bullying [7], although scholars have also identified some individual-related antecedents such as low self-esteem and poor social skills [8]. Studies thus showed that exposure to workplace bullying is a multicausal phenomenon [9]. However, these studies focusing on work- or individual-related antecedents have been developed independently of each other, although scholars underlined that the interaction between* both work- and individual-related antecedents* should be investigated to fully grasp the origin of exposure to workplace bullying [9]. In line with this suggestion, scholars claim that the effect of work stressors (i.e., work-related antecedents) on their outcomes could be influenced by coping strategies (i.e., individual-related antecedents) [10]. Despite these claims, studies investigating the interaction between work stressors and coping strategies to bullying are lacking [11].

In reply, this study aims to bridge the research lines on work-related* and* individual-related antecedents of workplace bullying by investigating the interaction between work stressors (i.e., workload, job insecurity, role conflict, and role ambiguity) and employees' coping strategies (i.e., problem- and emotion-focused) in association to exposure to workplace bullying. By investigating how the interaction between these factors may prevent or evoke exposure to workplace bullying, this study may additionally identify possible work- and individual-related prevention areas.

Studies have particularly underlined the negative impact of workload [12], job insecurity [13], role conflict, and role ambiguity [14] on exposure to workplace bullying. A recent systematic review showed that these work stressors are the most important antecedents of exposure to workplace bullying [11]. The association between those work stressors and exposure to bullying may be theoretically substantiated by the Work Environment Hypothesis [15] and the General Strain Theory [16]: a poor psychosocial work environment (i.e., work stressors) may trigger exposure to bullying because it depletes employees' energy, causing strain [16, 17]. Strained employees have difficulties in defending themselves against bullying acts and offer little resistance [17, 18]. Consequently, they become an “easy target” for exposure to workplace bullying [13].

The negative impact of work stressors on exposure to workplace bullying could be altered by coping strategies [10, 11]. In other words, employees' coping strategies could be potential moderators of the association between work stressors and exposure to bullying. The literature defines coping in at least two ways. Some studies conceptualise coping as fluctuating states depending on situational appraisals (i.e., state-like disposition) [19], while other studies found that the tendency to use certain coping strategies can be relatively stable over time and situations (i.e., trait-like disposition) [20, 21]. As the present study aims to investigate the interaction between work- and individual-related antecedents of exposure to workplace bullying, we align with the definition of coping strategies as a trait-like disposition. In this study, coping strategies refer to the* employees' tendency* to make cognitive and behavioural efforts to manage, tolerate, or reduce work stressors [10]. These coping strategies are either oriented at tackling the problem (“problem-focused”) or at managing emotions associated with the stressor (“emotion-focused”) [10]. Carver et al. [22] identified “active coping,” “planning,” and “seeking social support for instrumental reasons” as important problem-focused coping strategies, while “focus on and venting of emotions,” “behavioural disengagement,” “mental disengagement,” and “seeking social support for emotional reasons” were identified as emotion-focused coping strategies.

According to the Three-Way Model of Workplace bullying, work stressors may particularly trigger exposure to bullying when employees apply inefficient coping strategies, whereas applying efficient coping strategies may reduce exposure to bullying [23]. According to the pioneers in coping research, Lazarus and Folkman [10], emotion-focused coping strategies reduce the negative emotions associated with the stressor in the short term but may prevent employees from performing a suitable action to address the problem. Emotion-focused coping strategies may therefore impair employee well-being. This view is supported by previous studies indicating that “focus on and venting of emotions,” “behavioural disengagement,” “mental disengagement,” and “seeking social support for emotional reasons” are related to impaired well-being [e.g., [22, 24, 25]]. It also aligns with a recent review showing that using emotion-focused coping strategies as a dominant strategy is related to strain outcomes (e.g., emotional exhaustion and depersonalization) [26]. Emotion-focused coping strategies may thus be an inefficient way of coping with work stressors. Similarly, we propose that they may trigger exposure to workplace bullying: employees experiencing high levels of work stressors in combination with using inefficient coping strategies (i.e., emotion-focused coping strategies) tend to (unknowingly) breach well-established norms, habits, expectations, or values within their workplace [27]. For example, a stressed employee may look for distractions to avoid the problem and thus perform at a lower level than his/her colleagues. Colleagues may not accept that these norms are breached and may, in turn, try to restore the norms by punishing this employee or demonstrating negative acts towards them [Social Interactionist Theory; [23, 27, 28]]. Alternatively, a stressed employee may ventilate his/her emotions frequently to his/her colleagues, which may interfere with their work and hamper their performance. In reply, they may demonstrate negative acts towards the stressed employee for interfering with their work [Social Interactionist Theory; [23, 27, 28]]. In sum, we hypothesise the following.

**Hypothesis 1 .**

Emotion-focused coping strategies increase the association between work stressors, including workload* (H1a)*, job insecurity* (H1b)*, role conflict* (H1c)*, and role ambiguity* (H1d)*, and exposure to workplace bullying (i.e., amplifying effects).

In contrast, problem-focused coping strategies may be efficient in dealing with work stressors, as they are focused at solving the issue [10]. Previous studies have demonstrated that “active coping,” “planning,” and “seeking social support for instrumental reasons” were associated with positive health outcomes [e.g., [19, 22]] and were negatively correlated with strain outcomes, such as psychological symptoms and emotional exhaustion [26, 29]. Accordingly, we expect problem-focused coping strategies to decrease the association between work stressors and exposure to bullying: employees who cope with work stressors in a problem-focused way are putting effort into solving the problem instead of breaching valued norms, habits, expectations, or values [23, 27]. They gain control over the stressful situation by defining and interpreting the situation, planning solutions, and choosing a course of action which may avoid or reduce exposure to bullying [10, 30]. In sum, we hypothesise the following.

**Hypothesis 2 .**

Problem-focused coping strategies decrease the association between work stressors, including workload* (H2a)*, job insecurity* (H2b)*, role conflict* (H2c)*, and role ambiguity* (H2d)*, and exposure to workplace bullying (i.e., buffering effects).

## 2. Methods

### 2.1. Study Context and Participants

Cross-sectional data were collected from September until November 2014 by means of online and paper-and-pencil questionnaires distributed by an external service for optimising work environments (IDEWE). A total of 6,499 Flemish employees from 16 organisations in various sectors (i.e., healthcare, manufacturing, governmental, and service sectors) were invited to complete a questionnaire on psychosocial risk factors and work-related well-being [31]. All participants provided an informed consent that underlined the anonymity of their answers, stated that their participation was voluntary, and shared the researchers' contact information. The Social and Societal Ethics Committee (SMEC) of KU Leuven approved the study protocol (G-2014 07 025).

The final sample consisted of 3,105 Flemish employees (response rate of 48%) who completed the questionnaire. The mean age of the participants was 42 years (SD = 11.00). In total, 33% of the respondents were male, 68% had a full-time position, and 91% had a permanent contract. The participants were employed in healthcare (75%), manufacturing (9%), governmental (4%), and service (12%) sectors.

### 2.2. Measures

The variables were measured using established and internationally validated scales. The means, standard deviations, and correlations are presented in Table 1.

*Exposure to workplace bullying* (*α* = .85) was measured by means of the Short Negative Acts Questionnaire (S-NAQ) [32]. Respondents were asked to indicate how often they were confronted with a list of nine bullying acts during the last six months (e.g., “gossip or rumours about you”). The response categories ranged from “never” (=1) to “now and then” (=2), “monthly” (=3), “weekly” (=4), and “daily” (=5).

*Workload* (*α* = .87) was assessed using three items from the Questionnaire Experience and Evaluation of Work (QEEW) [33], including “I have to work extra hard in order to complete a task.”* Role ambiguity* (*α* = .82) was measured using three items from the Short Inventory to Monitor Psychosocial Hazards (SIMPH) [34]. An example of an item is “I know exactly what others expect of me in my work (R).”* Role conflict* (*α* = .79) was measured using three items of the Work Conditions and Control Questionnaire (WOCCQ; e.g., “I receive contradictory instructions”) [35].* Job insecurity* (*α* = .81) was measured by using three items from the scale by Vander Elst et al. [36], for example, “I think I might lose my job in the near future.” The items regarding the abovementioned work stressors were rated on a five-point Likert scale ranging from “almost never” (=1), “rather seldom” (=2), “sometimes” (=3), “often” (=4), and “almost always” (=5).

*Coping strategies* were assessed by 28 items from the COPE [22]. Following the idea that coping strategies represent individual factors expressing the tendency to apply certain strategies more than others, respondents were asked to indicate what they* usually* do when facing a stressful situation. The response categories varied from “almost never” (=1), “rather seldom” (=2), “sometimes” (=3), “often” (=4), and “almost always” (=5).* Problem-focused coping strategies* were measured with three subscales: four items tapped into* “active coping”* (e.g., “I concentrate my efforts on doing something about it”) and four into* “planning”* (e.g., “I think hard about what steps to take”), and another four measured* “seeking social support for instrumental reasons”* (e.g., “I try to get advice from someone about what to do”). The alpha coefficients for these scales were .83, .85, and .91, respectively*. Emotion-focused coping strategies* were measured using four subscales with four items each:* “focusing on and venting of emotions” *(e.g., “I get upset and show my emotions”),* “behavioural disengagement” *(e.g., “I just give up trying to reach my goal”),* “mental disengagement” *(e.g., “I turn to work or other substitute activities to take my mind off things”), and* “seeking social support for emotional reasons” *(e.g., “I get sympathy and understanding from someone”). Cronbach's alpha coefficients were .85, .86, .69, and .92, respectively.

Finally, age (years) and gender (0 = female, 1 = male) were measured.

### 2.3. Statistical Analyses

Analyses were performed with the software package AMOS 22. The construct validity of the scales was evaluated by means of Confirmatory Factor Analysis (CFA) [37]. The hypothesised measurement model contained 12 factors in which all items loaded on the corresponding latent variable (i.e., exposure to workplace bullying, workload, job insecurity, role conflict, role ambiguity, “active coping,” “planning,” “seeking social support for instrumental reasons,” “focusing on and venting of emotions,” “behavioural disengagement,” “mental disengagement,” and “seeking social support for emotional reasons”). We compared the measurement model with five alternative models: (1) a one-factor model in which all items were loaded on the same factor, (2) a four-factor model with general work stressors (i.e., the items of workload, job insecurity, role conflict, and role ambiguity), general problem-focused coping strategies (i.e., the items of “active coping,” “planning,” and “seeking social support for instrumental reasons”), general emotion-focused coping strategies (i.e., the items of “focusing on and venting of emotions,” “behavioural disengagement,” “mental disengagement,” and “seeking social support for emotional reasons”), and exposure to workplace bullying as latent factors, (3) a six-factor model with workload, job insecurity, role conflict, role ambiguity, general coping strategies (i.e., the items of “active coping,” “planning,” “seeking social support for instrumental reasons,” “focusing on and venting of emotions,” “behavioural disengagement,” “mental disengagement,” and “seeking social support for emotional reasons”), and exposure to workplace bullying as latent factors, (4) a seven-factor model with workload, job insecurity, role conflict, role ambiguity, general problem-focused coping strategies (i.e., the items of “active coping,” “planning,” and “seeking social support for instrumental reasons”), general emotion-focused coping strategies (i.e., the items of “focusing on and venting of emotions,” “behavioural disengagement,” “mental disengagement,” and “seeking social support for emotional reasons”), and exposure to workplace bullying as latent factors, and (5) a nine-factor model with general work stressors (i.e., the items of workload, job insecurity, role conflict, and role ambiguity), “active coping,” “planning,” “seeking social support for instrumental reasons,” “focusing on and venting of emotions,” “behavioural disengagement,” “mental disengagement,” “seeking social support for emotional reasons,” and exposure to workplace bullying as latent factors. In all models, the latent variables were allowed to covary. The *χ*^2^ difference test was used to compare the hypothesised measurement model with the alternative measurement models [37, 38]. The fit of the models was evaluated based on Comparative Fit Index (CFI), Tucker-Lewis Index (TLI), Root Mean Square Error of Approximation (RMSEA), and Standardized Root Mean Residual (SRMR) [38]. Values above .90 for CFI and TLI indicate a good fit, while values above .95 indicate an excellent fit [38, 39]. Values close to .08 for RMSEA and values close to .10 for SRMR indicate a relatively good fit between the measurement model and the observed data [38, 39]. Values below .05 for RMSEA and values below .09 for SRMR indicate an excellent fit [38].

In line with Bakker et al. [40] and following the procedure of Mathieu et al. [41, 42], we investigated the hypotheses by means of Moderated Structural Equation Modelling (MSEM). MSEM was used because it has the ability to (a) assess and correct for measurement error and (b) provide measures of fit of the models under investigation [37]. For each pair of a work stressor and a coping strategy, two models were tested and compared: (1) a model without an interaction factor and (2) a model with an interaction factor. In the model without the interaction, one of the four work stressors and one of the seven coping strategies were modelled as the exogenous factors and workplace bullying was the endogenous factor. To this model, a factor reflecting the interaction between the work stressor and the coping strategy was added (i.e., model with interaction factor). The interaction term was calculated by multiplying the centred scale scores for the respective work stressor and coping strategy [43]. In both models, the centred scale score for the respective variable indicated the exogenous factors. The exogenous factors were allowed to covary. The error variance of each indicator was set equal to the product of its variance and one minus its reliability [41, 42]. The paths from the exogenous factors to their indicator were calculated using the square roots of the scale reliabilities [40–42, 44]. The reliability of the interaction term was calculated using the formula as described in Cortina et al. [42].

The path coefficients were estimated and the fit of each model was evaluated using CFI, TLI, RMSEA, and SRMSR. The interaction effects were considered as significant when (a) the Unstandardized Path Coefficient (UPC) from the interaction term to the endogenous factor (i.e., exposure to workplace bullying) was statistically significant* and* (b) the *χ*^2^ difference test indicated that the model with the latent interaction factors fits the data better in comparison to the model without the latent interaction factor. As we tested the relationships in this study in a pairwise manner, a Bonferroni correction of *p* < .002 (instead of *p* < .05) was used.

## 3. Results

### 3.1. Construct Validity of the Measurement Model

Table 2 shows that the proposed 12-factor model fitted the data well and better than the five alternative models, providing evidence for the hypothesised dimensionality of the study scales. While the RMSEA and SRMR values pointed at an excellent model fit [45], the CFI and TLI values did not meet the strict standards for an excellent model fit. Nevertheless, these CFI and TLI values were comparable to what many others consider to represent adequate model fit [45].

### 3.2. Tests of the Hypotheses

**Figure 1:**
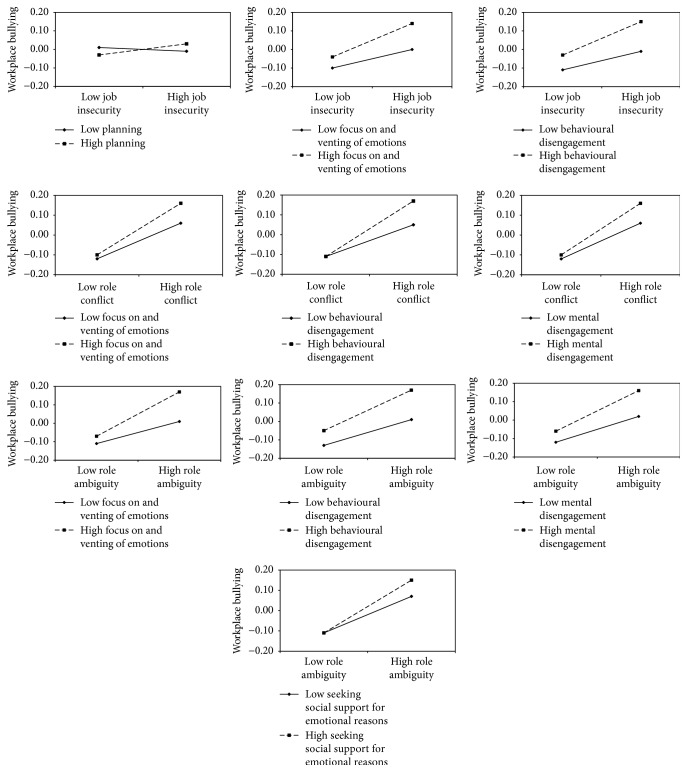
Plots of the significant interaction effects between work stressors and coping strategies in the prediction of exposure to workplace bullying.

Table 3 shows the results of the hypothesised moderating effects (information regarding the main effects of the investigated work stressors on exposure to workplace bullying can be retrieved by sending an e-mail to whitney.vandenbrande@idewe.be). Our first hypothesis was partially confirmed. Although we found no evidence for the moderating role of emotion-focused coping strategies in the association between workload and exposure to workplace bullying, some emotion-focused coping strategies moderated the association of job insecurity, role conflict, and role ambiguity with exposure to bullying. For these tests, the UPCs were significant for a Bonferroni correction of *p* < .002 and the models with the interaction term fitted the data significantly better than the models without an interaction term. In line with our expectations, plots of the significant interaction effects revealed amplifying effects of emotion-focused coping strategies (Figure 1). Specifically, employees using “focus on and venting of emotions” or “behavioural disengagement” when experiencing job insecurity, role conflict, and role ambiguity were more likely to be exposed to bullying. Similar results were found for employees using “mental disengagement” in the case of role conflict and role ambiguity and for employees using “seeking social support for emotional reasons” in the case of role ambiguity.

Our second hypothesis was rejected, as problem-focused coping strategies did not buffer the association between the work stressors (i.e., workload, job insecurity, role conflict, and role ambiguity) and exposure to workplace bullying. Although for some interactions the models with the interaction term fitted the data significantly better, the UPCs were not significant (*p* > .002). Notably, employees using “planning” strategies when experiencing job insecurity were more likely to be exposed to bullying (see Figure 1).

As the demographic variable of gender (0 = female; 1= male) was positively correlated with exposure to workplace bullying (Table 1), we reran all 28 pairwise models also controlling for gender. However, these analyses did not alter our conclusions. Age was not associated with exposure to workplace bullying and was therefore not included in this analysis.

## 4. Discussion

To our knowledge, this is the first study that investigates the moderating role of problem- and emotion-focused coping strategies in the association between work stressors and exposure to workplace bullying.

The results provided partial support for our first hypothesis on the amplifying effects of emotion-focused coping strategies in the association between work stressors and exposure to workplace bullying. The strengths of all the interaction effects were of similar size and rather small, based on the magnitude of the UPCs observed. First, most interaction effects were found for “focus on and venting of emotions” and “behavioural disengagement.” When experiencing job insecurity, role conflict, or role ambiguity, employees using these emotion-focused coping strategies were more likely to be exposed to bullying, in comparison with employees not using these strategies. Second, two interaction effects were found for “mental disengagement.” Employees with the tendency to use “mental disengagement” in the case of role conflict or role ambiguity were more likely to be exposed to bullying. Finally, one interaction effect was found for “seeking social support for emotional reasons.” Employees with the tendency to use “seeking social support for emotional reasons” in the case of role ambiguity were more likely to be exposed to workplace bullying.

From an empirical perspective, these results align with previous studies on coping and strain outcomes. For example, a longitudinal study showed that emotion-focused coping strategies amplified the negative impact of role conflict on emotional exhaustion [46]. Moreover, Chen and Kao [47] found evidence for emotion-focused coping strategies as an amplifier in the association between job hassles and burnout. From a theoretical perspective, it seems that applying emotion-focused coping strategies in combination with* specific* work stressors (i.e., job insecurity, role conflict, or role ambiguity) makes employees more vulnerable to bullying. According to the Three-Way Model of Workplace bullying and to Social Interactionism, employees may unknowingly breach habits and values within their organisation making them “easy” targets for workplace bullying [23, 27].

Notably, these results contradict recent suggestions in the work stress literature differentiating work stressors in terms of job hindrances and job challenges. In the literature, job hindrances (i.e., role conflict, role overload, and job insecurity) are defined as work stressors that are uncontrollable obstacles that hinder optimal functioning [48, 49]. Job challenges (i.e., workload) are work stressors that require some energy, but are nonetheless stimulating and help in achieving goals [48, 49]. The challenges-hindrances literature assumes that emotion-focused coping strategies are not helpful in reducing the potential negative impact of job challenges: as job challenges are perceived as controllable and may be helpful in achieving goals, using problem-focused coping strategies would be more beneficial [49, 50]. In contrast, as job hindrances are uncontrollable, emotion-focused coping strategies are more appropriate to be used to reduce the negative impact of job hindrances, while problem-focused coping strategies are assumed to increase their negative impact [49, 50]. Our findings, however, show that emotion-focused coping strategies amplify rather than buffer the association between job hindrances (i.e., job insecurity, role conflict, and role ambiguity) and exposure to workplace bullying. They thus contradict recent arguments in the work stress literature but are in line with the well-established view of the Three-Way Model of Workplace bullying [23] and Lazarus and Folkman [10].

Contrary to our expectations, no interaction effects between workload and the investigated emotion-focused coping strategies were found. Thus, this finding contradicts recent developments in the work stress literature arguing that emotion-focused coping strategies would be problematic in dealing with job challenges [49, 50]. Future research should investigate a wider range of coping strategies that would be relevant for workload, such as cognitive reframing [51]. Cognitive reframing might be a more efficient coping strategy than the other investigated coping strategies, as it may, for example, influence the way employees perceive workload. By applying cognitive reframing as a coping strategy, the situation may become less stressful: it may change the perception of the initial stressors in a way that may reduce the perceived workload [52].

Our second hypothesis was rejected: we found no evidence for the buffering role of problem-focused coping strategies. Moreover, in contrast to our expectations, “planning” (i.e., a problem-focused coping strategy) amplified rather than buffered the association between job insecurity and exposure to workplace bullying. Employees using “planning” to deal with job insecurity were more likely to be exposed to bullying. Although unexpected, this finding aligns with previous results showing that problem-focused coping in combination with job insecurity is associated with negative outcomes in terms of low job satisfaction and high turnover intention [53]. Our results extend those findings to being exposed to workplace bullying. From a theoretical perspective, our findings can be explained through the work of Folkman et al. [19] who state that the efficiency of coping strategies depends on the source of the stressor. Problem-focused coping strategies are more efficient when the source of the stressor is clear or controllable [10]. In the case of job insecurity, the source of the uncertain environment is unclear, and employees often are not able to control or handle the economic status of their company [53]. This also aligns with the challenges-hindrance literature arguing that problem-focused coping strategies are less effective and thus increase the negative impact of job hindrances (i.e., job insecurity) on strain outcomes (i.e., workplace bullying), as described earlier [49, 50]. As the efficiency of a coping strategy may depend on how well it fits with a particular stressor [51], further research is needed to investigate* specific combinations of work stressors and coping strategies* to determine which strategies are more appropriate to prevent exposure to workplace bullying.

### 4.1. Limitations and Paths for Future Research

Some limitations should be considered in interpreting the findings of this study. First, this study has a cross-sectional research design. Consequently, the conclusions do not allow us to determine the direction of the predicted associations. However, our research model was based on multiple previous longitudinal studies that already identified causal (cross-lagged) relationships from work stressors to exposure to workplace bullying rather than the other way around [54, 55]. Moreover, cross-sectional data might be appropriate to investigate interaction effects, because the moderator is not part of a causal sequence but qualifies an association between variables [56]. Nevertheless, we advise future studies to use a longitudinal design to replicate our findings and investigate the moderating role of coping strategies in the* lagged *relationship from work stressors to exposure to workplace bullying. Notably, as workplace bullying can also be considered a social stressor [e.g., [57]], it may be interesting to investigate the moderating role of coping strategies in the lagged relationship from exposure to workplace bullying to strain. This aligns with Lazarus and Folkman [10], equally suggesting that coping strategies may influence the impact of workplace bullying on its outcomes. As mentioned above, the authors state that the efficiency of coping strategies depends on the controllability of the perceived stressor (i.e., workplace bullying) [10]. Moreover, Lazarus and Folkman [10] propose that problem-focused coping strategies are efficient when the stressor is perceived as controllable, while emotion-focused coping strategies are expected to be efficient when the stressor is perceived as uncontrollable [58]. Exposure to workplace bullying is typically defined as uncontrollable: we thus expect that emotion-focused coping strategies would reduce this association, while problem-focused coping strategies are expected to amplify this association [59]. This theoretical reasoning aligns with recent findings from previous studies investigating these hypotheses [e.g., [59]]. However, it would be interesting to examine if the proposed associations between work stressors and coping strategies are the same for employees exposed to workplace bullying as compared to employees not exposed to bullying.

Second, due to the use of self-reported measures common method bias may have inflated the associations between our study variables [60]. However, self-reported measures are appropriate in this study because we aimed to investigate the way employees (a)* perceived* work stressors, (b)* preferred* the use of certain coping strategies, and (c)* perceived or experienced* acts of workplace bullying. Additionally, self-reported measures are dominantly used in research on workplace bullying [61]. We attempted to reduce the risk of common method bias by emphasizing the voluntary nature of this study and the anonymous treatment of the study results and by demonstrating the construct validity of the study scales in a series of CFAs. Nevertheless, future research should consider using multisource data to avoid problems with common method bias.

Third, as we used pairwise tests and the same relationships were tested repeatedly, a Bonferroni correction with *p* < .002 (instead of *p* < .05 or *p* < .01) was used. This correction may have led to conservative conclusions: several hypotheses were rejected at the .002 level but could be accepted at the .05 level (e.g., the interaction between workload and behavioural disengagement) or at the .01 level (e.g., the interaction between workload and seeking support for emotional reasons). Nevertheless, by applying a Bonferroni correction, we reduced the risk of Type Ι errors [62]. However, because of the relative large sample size, this is much less an issue in this study [63].

Fourth, our study sample did not represent all sectors. For example, employees working in the education sector and construction industry were not included in our sample. Furthermore, employees working in the health care sector were overrepresented (75%). Therefore, researchers should be careful about generalising our conclusions to employees working in all sectors. However, we do not believe that the sample composition would affect our results and that using a more representative sample would have led to other results [64]. Previous research found no differences regarding exposure to workplace bullying between health care workers and employees working in other sectors [65].

Fifth, it would be interesting to investigate the moderating role of coping strategies in the association between other antecedents and exposure to workplace bullying. For example, a prospective study showed that mental distress predicts exposure to workplace bullying, showing that individual characteristics may make employees more vulnerable to bullying [66]. Following the results of our study, it would be interesting to also investigate the moderating role of problem- and emotion-focused coping strategies (i.e., individual-related factors) in the association between mental distress and exposure to workplace bullying to examine whether individual factors may also be a risk factor of becoming bullied.

Finally, this study focused on targets of exposure to workplace bullying. However, future studies should investigate the moderating role of coping strategies in the association between work stressors and workplace bullying from the perspective of the perpetrator. Indeed, high levels of work stressors in combination with inefficient coping strategies may produce irritation and hostility, which may result in demonstrating negative acts towards coworkers. This view aligns with the Frustration-Aggression hypothesis [67]: when dealing with frustrations and the accompanying negative emotions, employees may act out these frustrations through negative actions [67]. This process can be amplified when using inefficient coping mechanisms because these employees do not reduce the antecedent conditions which cause these frustrations. As a result they become more frustrated and demonstrate negative acts towards other colleagues. Future research is needed to explore this hypothesis.

## 5. Conclusion

This study investigated the moderating role of employees' problem- and emotion-focused coping strategies in the association between work stressors and exposure to workplace bullying. As expected, some emotion-focused coping strategies amplified the association between work stressors and exposure to bullying. However, we found no evidence for the buffering role of problem-focused coping strategies in the association between work stressors and being bullied. Based on our results, we advise organisations to implement interventions that focus on making employees aware of the possible amplifying effects of emotion-focused coping strategies when they are experiencing job insecurity, role conflict, and/or role ambiguity. We advise future research to investigate specific combinations of different (types of) work stressors and coping strategies to determine which coping strategies are efficient in preventing workplace bullying.

## Figures and Tables

**Table 1:** Means, standard deviations, and correlations (*N* = 3,105).

	*M*	SD	(1)	(2)	(3)	(4)	(5)	(6)	(7)	(8)	(9)	(10)	(11)	(12)	(13)	(14)
(1) Age	41.61	11.00	—	.13^*∗∗*^	.05^*∗*^	−.09^*∗∗*^	−.04	−.13^*∗∗*^	.03	.10^*∗∗*^	−.10^*∗∗*^	−.09^*∗∗*^	.05^*∗*^	−.09^*∗∗*^	−.18^*∗∗*^	−.02
(2) Male	n.a.	n.a.		—	−.02	.01	.08^*∗∗*^	.09^*∗∗*^	−.08^*∗∗*^	.02	−.12^*∗∗*^	−.25^*∗∗*^	.03	−.08^*∗∗*^	−.35^*∗∗*^	.06^*∗∗*^
(3) Workload	3.42	0.84			—	.10^*∗∗*^	.43^*∗∗*^	.17^*∗∗*^	.07^*∗∗*^	.05^*∗∗*^	−.02	.13^*∗∗*^	.04^*∗*^	.09^*∗*^	.08^*∗∗*^	.22^*∗∗*^
(4) Job insecurity	2.09	0.90				—	.27^*∗∗*^	.25^*∗∗*^	−.07^*∗∗*^	−.08^*∗∗*^	−.02	.13^*∗∗*^	.16^*∗∗*^	.14^*∗∗*^	.02	.29^*∗∗*^
(5) Role conflict	2.41	0.91					—	.42^*∗∗*^	−.07^*∗∗*^	−.05^*∗∗*^	−.03	.17^*∗∗*^	.23^*∗∗*^	.20^*∗∗*^	.02	.46^*∗∗*^
(6) Role ambiguity	1.93	0.73						—	−.16^*∗∗*^	−.10^*∗∗*^	−.08^*∗∗*^	.13^*∗∗*^	.16^*∗∗*^	.13^*∗∗*^	−.01	.33^*∗∗*^
(7) Active coping	4.02	0.61							—	.63^*∗∗*^	.35^*∗∗*^	−.08^*∗∗*^	−.30^*∗∗*^	−.08^*∗∗*^	.12^*∗∗*^	−.04^*∗*^
(8) Planning	3.71	0.76								—	.39^*∗∗*^	−.08^*∗∗*^	−.26^*∗∗*^	−.08^*∗∗*^	.10^*∗∗*^	−.03
(9) SOCINSTR	3.44	0.87									—	.18^*∗∗*^	−.07^*∗∗*^	.06^*∗∗*^	.38^*∗∗*^	−.03
(10) VENT	2.22	0.80										—	.38^*∗∗*^	.36^*∗∗*^	.43^*∗∗*^	.19^*∗∗*^
(11) BD	1.70	0.71											—	.42^*∗∗*^	.04^*∗*^	.22^*∗∗*^
(12) MD	2.36	0.75												—	.27^*∗∗*^	.19^*∗∗*^
(13) SOCEMO	3.08	0.98													—	.06^*∗∗*^
(14) EWB	1.48	0.51														—

*Note.* n.a.: not applicable; SOCINSTR: seeking social support for instrumental reasons; VENT: focus on and venting of emotions; MD: mental disengagement; BD: behavioural disengagement; SOCEMO: seeking social support for emotional reasons; EWB: exposure to workplace bullying; ^*∗*^*p* < .05; ^*∗∗*^*p* < .01.

**Table 2:** Results of Confirmatory Factor Analysis (*N* = 3,105).

Model	Latent factors	*χ* ^2^	df	CFI	TLI	RMSEA	SRMR	Model comparison	Δ*χ*^2^	Δdf
(1) 12-factor model	WL, JI, RC, RA, AC, PL, SOCINSTR, VENT, MD, BD, SOCEMO, EWB	7009.54^*∗∗∗*^	1061	.92	.92	.04	.04	/	/	/

(2) One-factor model	General factor	62958.46^*∗∗∗*^	1127	.21	.18	.13	.15	4 versus 1	55948.92^*∗∗∗*^	66

(3) Four-factor model	Stressors, PFC, EFC, EWB	38167.12^*∗∗∗*^	1121	.53	.51	.10	.11	5 versus 1	31157.58^*∗∗∗*^	60

(4) Six-factor model	WL, RA, JI, RC, General coping, EWB	39075.20^*∗∗∗*^	1112	.52	.49	.11	.13	6 versus 1	32065.66^*∗∗∗*^	51

(5) Seven-factor model	WL, JI, RC, RA, PFC, EFC, EWB	29109.328^*∗∗∗*^	1106	.64	.62	.09	.10	3 versus 1	22099.79^*∗∗∗*^	45

(6) Nine-factor model	Stressors, AC, PL, SOCINSTR, VENT, MD, BD, SOCEMO, EWB	16145.45^*∗∗∗*^	1091	.81	.79	.07	.06	2 versus 1	9135.91^*∗∗∗*^	30

*Note*. WL: workload; RA: role ambiguity; JI: job insecurity; RC: role conflict; PFC: problem-focused coping; EFC: emotion-focused coping; EWB: exposure to workplace bullying; AC: active coping; PL: planning; SOCINSTR: seeking social support for instrumental reasons; VENT: focus on and venting of emotions; MD: mental disengagement; BD: behavioural disengagement; SOCEMO: seeking social support for emotional reasons; ^*∗∗∗*^*p* < .001.

**Table 3:** Results of Moderated Structural Equation Modelling analyses for the interaction between work stressors and coping strategies (*N* = 3,105).

Interaction effect	UPC	SE	SPC	*χ* ^2^	CFI	TLI	RMSEA	SRMR	Model comparison	
									Δ*χ*^2^	Δdf
*Active coping:*										
Workload × active coping	.003	.007	.011	1099.732^*∗∗∗*^	.894	.868	.080	.048	82.209^*∗∗∗*^	10
Job insecurity × active coping	.011	.007	.036	871.594^*∗∗∗*^	.917	.896	.071	.040	30.760^*∗∗∗*^	10
Role conflict × active coping	.007	.006	.024	1232.564^*∗∗∗*^	.888	.860	.085	.050	17.594	10
Role ambiguity × active coping	.006	.008	.016	1034.238^*∗∗∗*^	.903	.879	.077	.045	29.625^*∗∗∗*^	10
*Planning:*										
Workload × planning	.007	.006	.026	1056.502^*∗∗∗*^	.898	.873	.078	.046	19.927^*∗*^	10
Job insecurity × planning	.021^b^	.007	.073	888.747^*∗∗∗*^	.915	.894	.071	.040	33.602^*∗∗∗*^	10
Role conflict × planning	.012	.005	.052	1259.622^*∗∗∗*^	.886	.858	.086	.051	32.901^*∗∗∗*^	10
Role ambiguity × planning	.012	.007	.037	1071.857^*∗∗∗*^	.899	.874	.079	.047	58.995^*∗∗∗*^	10
*Seeking social support for instrumental reasons:*										
Workload × seeking social support for instrumental reasons	.007	.005	.028	1058.567^*∗∗∗*^	.898	.873	.078	.047	15.851	10
Job insecurity × seeking social support for instrumental reasons	.008	.005	.036	876.077^*∗∗∗*^	.916	.895	.071	.039	9.203	10
Role conflict × seeking social support for instrumental reasons	.008	.004	.039	1250.407^*∗∗∗*^	.886	.858	.085	.050	13.735	10
Role ambiguity × seeking social support for instrumental reasons	.010	.006	.037	1052.848^*∗∗∗*^	.900	.876	.078	.047	36.766^*∗∗∗*^	10
*Focus on and venting of emotions:*										
Workload × focus on and venting of emotions	.002	.005	.007	1055.240^*∗∗∗*^	.900	.875	.078	.047	35.610^*∗∗∗*^	10
Job insecurity × focus on and venting of emotions	.021^*∗∗∗*^	.005	.089	885.721^*∗∗∗*^	.916	.896	.071	.040	42.092^*∗∗∗*^	10
Role conflict × focus on and venting of emotions	.023^*∗∗∗*^	.005	.104	1254.362^*∗∗∗*^	.888	.860	.085	.051	37.986^*∗∗∗*^	10
Role ambiguity × focus on and venting of emotions	.032^*∗∗∗*^	.008	.101	1022.669^*∗∗∗*^	.904	.881	.077	.045	22.121^*∗*^	10
*Mental disengagement:*										
Workload × mental disengagement	.005	.006	.022	1038.441^*∗∗∗*^	.901	.877	.077	.046	17.059	10
Job insecurity × mental disengagement	.017	.005	.074	858.832^*∗∗∗*^	.919	.899	.070	.039	12.695	10
Role conflict × mental disengagement	.018^*∗∗∗*^	.005	.085	1241.448^*∗∗∗*^	.889	.862	.085	.050	27.217^*∗∗*^	10
Role ambiguity × mental disengagement	.023^b^	.007	.079	1047.318^*∗∗∗*^	.902	.878	.078	.046	44.578^*∗∗∗*^	10
*Behavioural disengagement:*										
Workload × behavioural disengagement	.012	.006	.042	1044.847^*∗∗∗*^	.901	.876	.078	.046	11.138	10
Job insecurity × behavioural disengagement	.020^*∗∗∗*^	.006	.075	949.500^*∗∗∗*^	.911	.889	.074	.044	96.242^*∗∗∗*^	10
Role conflict × behavioural disengagement	.027^*∗∗∗*^	.005	.110	1323.539^*∗∗∗*^	.883	.854	.088	.055	100.071^*∗∗∗*^	10
Role ambiguity × behavioural disengagement	.024^*∗∗∗*^	.007	.074	1074.327^*∗∗∗*^	.900	.876	.079	.049	63.595^*∗∗∗*^	10
*Seeking social support for emotional reasons:*										
Workload × seeking social support for emotional reasons	.001	.005	.003	1062.705^*∗∗∗*^	.898	.873	.078	.047	25.580^*∗∗*^	10
Job insecurity × seeking social support for emotional reasons	.009	.004	.047	866.678^*∗∗∗*^	.917	.897	.070	.039	8.944	10
Role conflict × seeking social support for emotional reasons	.013	.005	.057	1246.383^*∗∗∗*^	.887	.859	.085	.049	18.30	10
Role ambiguity × seeking social support for emotional reasons	.018^*∗∗∗*^	.005	.074	1034.948^*∗∗∗*^	.902	.878	.077	.046	22.219^*∗*^	10

*Note.* UPC: unstandardized path coefficient; SE: standard error; SPC: standardized path coefficient; Model Comparison included comparing the fit of the model with interaction term and the model without interaction term; ^*∗*^*p* < .05; ^*∗∗*^*p* < .01; ^*∗∗∗*^*p* < .001; ^b^*p* < .002.
